# Indoor localisation through object detection within multiple environments utilising a single wearable camera

**DOI:** 10.1007/s12553-016-0159-x

**Published:** 2016-12-22

**Authors:** Colin Shewell, Chris Nugent, Mark Donnelly, Haiying Wang, Macarena Espinilla

**Affiliations:** 1 Ulster University, Shore Road, Newtownabbey, Co. Antrim, BT37 0QB UK; 2 Universidad de Jaén, Campus Las Lagunillas, s/n, 23071 Jaén, Spain

**Keywords:** Ageing in place, Ambient assisted living, Context-aware services, Machine vision, Wearable computing

## Abstract

The recent growth in the wearable sensor market has stimulated new opportunities within the domain of Ambient Assisted Living, providing unique methods of collecting occupant information. This approach leverages contemporary wearable technology, Google Glass, to facilitate a unique first-person view of the occupants immediate environment. Machine vision techniques are employed to determine an occupant’s location via environmental object detection. This method provides additional secondary benefits such as first person tracking within the environment and lack of required sensor interaction to determine occupant location. Object recognition is performed using the Oriented Features from Accelerated Segment Test and Rotated Binary Robust Independent Elementary Features algorithm with a K-Nearest Neighbour matcher to match the saved key-points of the objects to the scene. To validate the approach, an experimental set-up consisting of three ADL routines, each containing at least ten activities, ranging from drinking water to making a meal were considered. Ground truth was obtained from manually annotated video data and the approach was previously benchmarked against a common method of indoor localisation that employs dense sensor placement in order to validate the approach resulting in a recall, precision, and F-measure of 0.82, 0.96, and 0.88 respectively. This paper will go on to assess to the viability of applying the solution to differing environments, both in terms of performance and along with a qualitative analysis on the practical aspects of installing such a system within differing environments.

## Introduction

The remarkable increase in life expectancy can be viewed as one of the greatest achievements of the 20th century. As a result the oldest (aged 65 plus) in society are now regarded as the most rapidly expanding group within the population [12]. This has resulted in a surge in the increasing numbers of age related conditions, such as dementia and general cognitive decline associated with ageing. One solution to address the care provision required by these is postulated to involve technology based smart environments that have the ability to support ageing-in-place, otherwise known as Ambient Assisted Living (AAL). This solution aims to afford inhabitants the ability to remain within their own home for longer, and to maintain an acceptable level of quality of life. Thereby delaying the requirement to be re-situated within full time care facilities [12].

Over recent years ‘smart’ technologies for use within smart homes have gained increasing usage and acceptance, in particular, due to the widespread adoption of smart-phones along with the introduction of wearable technology to the consumer market. This has stimulated new opportunities within the domain of pervasive computing, particularly with the advent of head-mountable wearables such as Google Glass, SmartEyeglass, and the M100. These provide a unique ability to obtain a first-person view of an occupant’s activities and their environment.

This paper proposes a solution to facilitate indoor localisation through the use of a single ‘always on’ wearable camera, which has been implemented using the Google Glass platform. Occupant location is determined using machine vision techniques that identify reference objects located within the environment which are then cross-referenced against a knowledge base that contains the objects known location.

The remainder of the paper is structured as follows. Section 2 outlines related work within the field of indoor localisation, focusing on those that use machine vision techniques. Section 3 discusses the methodology used, presenting an overview of the system in addition to more detailed information regarding the feature point detection and matching algorithms used. Along with a description of the routines used to carry out the experiment. Section 4 presents and discusses the results that are also benchmarked against a dense sensor solution along with a comparison of the results of the Jaèn [27] and UU labs [28]. Section 5 offers a discussion between the results from the UU an Jaèn experiments along with a qualitative analysis of the practical aspects of installing such a system in a home in comparision to traditional systems. Finally Section 6 provides a set of conclusions that critique these early findings and outlines the plans for future work.

## Related work

This Section presents a summary of the current state-of-the-art of machine vision based solutions that facilitate indoor localisation. A general overview of indoor localisation methods are presented along with a number of studies which have a focus on applying contemporary technology using machine vision techniques within the domain of AAL. The findings are promising, however, several challenges are highlighted which will need to be addressed. Dense sensor solutions are also reviewed to provide a basis for benchmarking the proposed system.

There are multiple other methods of performing indoor occupant localisation, some of the methods that have previously been employed are RF/WiFi signals, and machine vision methods. RF and WiFi employ a similar method to obtaining the occupant location where the occupant carries a small device on their person, such as a smart phone, and the relative signal strength from broadcasting devices is measured. By measuring the signal strength from the broadcast devices, such as wireless access points, the occupants location can be determined [4]. A popular methods of indoor localisation through machine vision is the use of static cameras placed within the environment. Static cameras allows the use of machine methods, such as background subtraction, to ‘follow’ an occupant throughout the environment. There are, however, some limitations to this method, it may require multiple cameras to be placed in each room in order to cover the entire room. There is also the problem of occlusion, where the occupant may be wholly or partially blocked by items in the room, such as large items of furniture [9]. An additional method of machine vision is through the use of technology such as the Microsoft Kinect [14] which can detect occupants and when paired with frameworks such as the Controller Application Communication (CAC) framework [13] can allow the occupants location to be determined.

Okeyo et al. developed a dense sensor based solution incorporating multi-agents in order to provide services to occupant’s within smart homes [19]. Sensors were placed on specific objects that the user would interact with which would then record the time and location associated with that sensor in order to build contextual information. While the overall results were high (1.00, 0.88, 0.88 for precision, recall, and accuracy, respectively) it still suffers from the inherent problems that exist with dense sensor based methods, such as multiple occupancy and the need for sensor interaction. Along with the problem of the cost of installation, both in terms of financial costs but also the personal cost of having the system installed in an occupants home due to the time taken to perform the installation and the invasion of privacy as the equipment is installed in the occupants own home.

Rahal et al. implemented a system using anonymous dense sensor placement along with Bayesian filtering in order to determine occupant location [23]. The system was tested using a scenario of an occupants daily routine, the routine was performed by 14 subjects, one at a time. The system showed a mean localisation accuracy of 0.85, as the authors note however the system is only capable of supporting a single occupant [23] within a fixed environment.

Leotta and Mecalla [15] developed PLaTHEA (*P*eople *L*ocalization *a*nd *T*racking for *H*om*E* *A*utomation). PLaTHEA is a machine vision based system that acquires a stereo video stream from two network attached cameras in order to provide support for AAL. Two cameras are placed in each room, working in stereo, in order to ensure that as much of the room is covered and that occlusions are reduced. Foreground extraction is then performed in order to determine if occupants are present in the scene. PLaTHEA also performs identity recognition through the use of facial recognition. Nevertheless, there are some limitations to the PLaTHEA system, an issue that was identified by the authors, were when the system was monitoring a room with a wall greater than 10 metres then it was not possible to monitor without the use of costly acquisition hardware [15]. While the issue of cost is being addressed there is also the additional cost of having to install multiple cameras within each room that support is provided within. There is also the issue of multiple occupancy, due to the use of foreground extraction to identify occupants, while this is partially mitigated through the use of facial recognition it also requires that all the occupants are known and have SIFT features saved within the system [15]. There is also the additional problem of the Haar classifier being reliant on the occupants eye’s being clearly seen by the camera as this method of face detection will usually fail if the eyes are occluded [31].

Rivera-Rubio et al. [24] developed a system that estimated the user’s location through scene recognition. The experiment was carried out using an LG Google Nexus 4 and Google Glass. A dataset was gathered of the locations by recording a video of the occupant walking through the location ten times whilst wearing a recording device (50 % split between the Nexus 4 and Glass). This included a combination of day/night acquisitions and occasional strong lighting from windows. The system was tested using multiple descriptor methods (three custom designed and three standard methods) following a standard bag-of-words and kernel encoding pipeline, with HOG3D matching used as a baseline [24]. Results show errors as low as 1.6 metres over a 50 metre distance were achieved, however, for the purposes of AAL a greater level of refinement is required in order to distinguish where in a room the occupant is located and if possible what they are interacting with in order to provide relevant support. There is also the additional challenge of having to train the system to each environment that it is to be deployed within.

Zhang et al. [33] proposed a method of indoor location using still images captured at intervals from a smart-phone worn on a lanyard. This system has the goal of assisting those with impaired vision to navigate within an indoor environment. The system relies on collecting map data of a building, that describe features/descriptors along with their 3D co-ordinates, floor plans, and other location data. Images are then captured and sent at intervals from the smart-phone to a server for processing. Images are then matched against the template map of the building in order to determine location and offer directions should the user require them. Whilst this system works well for its intended use there are limitations when applied to an AAL situation. One problem, that the authors noted, was that there were null spots, were there was not enough features to create a map image, such as when the user makes a 90° turn, for example in a hall way or entering a room [33]. One other possible issue for an AAL application is that of the intermittent image capture that may result in missing key information, such as a room transition or an interaction with an appliance, which could be vital for context.

Orrite et al. [20] developed a system entitled ‘Memory Lane’ with the goal of providing a contextualised life-blog for those with special needs. It chronologically tagged and ordered images and sounds perceived by the user in order to provide contextual meaning. A data-set of images of the occupant’s environment was gathered and SIFT with RANSAC applied to obtain feature points. During each RANSAC iteration a candidate fundamental matrix was calculated using the eight-point algorithm [5], normalising the problem to improve robustness to noise. Their system consisted of a wearable camera that systematically recorded still images as the occupant moved throughout the environment which would then be matched against the previously collected image data-set of the environment. A feature match correspondence was used to established the distance of the occupant from the object. This involves generating a variable circle centred on the average position of the detected features and comparing it to the average position in the next image. If the radius increases it can be determined that the occupant is moving closer to the object. Some limitations of this solution are the need to gather the data-set of the environment along with the inherent problems with intermittent image gathering.

The presented system will use a head-mounted wearable camera streaming a live video feed, this should reduce occlusions and hope to reduce missing key information that an intermittent system may produce. Along with a greater refinement in the user’s location to assist in providing increasingly timely and relevant support. The proposed system will also not require any training for the environment that it is to be deployed within. The use of unique fiducial markers to identify common objects allows the system to be installed in multiple environments without the need to train to that environments unique layout.

## Methodology

This paper proposes a solution to facilitate indoor localisation through the use of a single ‘always on’ wearable camera, which has been implemented using the Google Glass platform. Occupant location is determined using machine vision techniques that identify reference objects located within the environment which are then cross-referenced against a knowledge base that contains the objects known location. The objects are identified through the use of fiducial markers placed on ‘key’ objects throughout the environment, within the context of the work presented fiducial markers are defined as images or scenes within the environment that support the alignment, identification, and tracking of objects or location [21]. In the proposed work the markers are placed on fixed objects in order to determine the position of a moving camera which will be worn on the occupant of interest. An example of this would be the sofa in a living room, if the sofa is detected we can determine that the occupant is in the living room and thus can provide the relevant support that may be needed within their immediate environment. The use of fiducial markers also alleviates the problem of trying to distinguish between multiple identical objects that may be within a household, such as the kitchen cabinets, as well as negating the need to recognise various models of appliances that may differ in their appearance. As the markers can be retro-fitted to any object the use of a smart environment is not required, therefore greatly reducing the cost of applying such a system to occupants own homes.

**Fig. 1:**
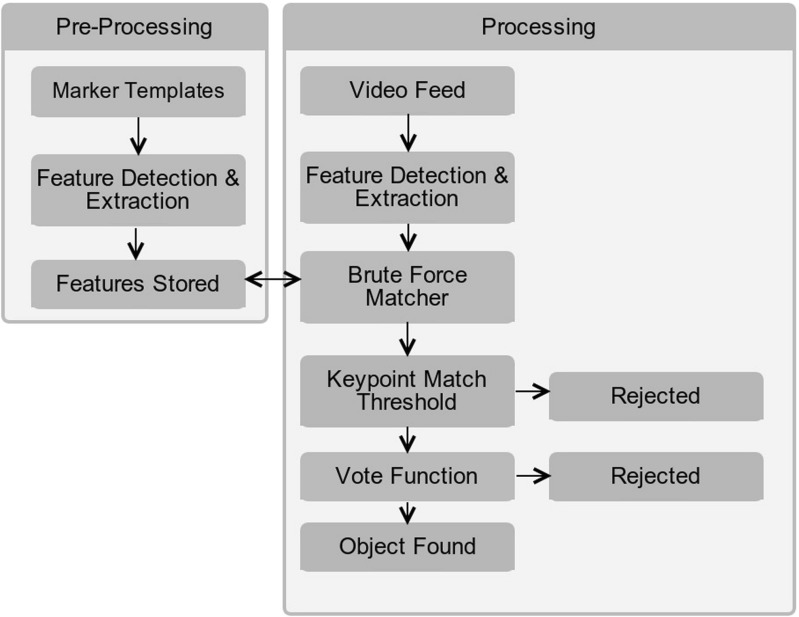
High level overview of machine vision system processing - consisting of a pre-processing section and a real-time processing section

Our proposed approach employs off-the-shelf machine vision tools to facilitate the detection of objects. Specifically the OpenCV Oriented FAST and Rotated BRIEF (ORB) algorithm for feature detection and descriptor extraction have been used. This is paired with a Brute-Force matcher to determine when the object of focus is present in the video stream. It is hypothesised that the use of a single wearable camera to determine the inhabitant’s location may facilitate inhabitant tracking within an environment. This may be used to provide enhanced contextual information based on their location. This approach has the advantage of reducing the set-up costs associated with alternative location tracking approaches, such as dense sensor placement [11]. This is achieved using machine vision techniques to identify reference objects within the patients field of view that are then cross-referenced against a knowledge base which indicates the room that the objects are located within. A high level overview of the process is shown in Fig. 1, consisting of a pre-processing section where the marker templates are learned and the real-time processing section where the learned templates are matched against the real-time video feed in order to provide marker/object detection. The system was tested in the Smart Environment Research Group (SERG) smart living space which consisted of a fully sensorised kitchen and living room [18]. The environment contains a suite of sensor technology, including PIR sensors, contact sensors, and floor pressure sensors. The presented method was benchmarked against a dense binary sensor deployment consisting of 14 individual sensors.

The vision aspect of the experiment was implemented using the Google Glass Explorer platform which allowed the recording of video (up to 1280x720) as well as natural spoken language commands. Relevant information can also be displayed via the prism display that is located over the right eye. The onboard processing consists of 682 MB useable RAM (1 GB RAM total – 342 MB reserved), and a dual core TI OMAP 4430 1GHz processor.

In order to assess the viability of applying the system to multiple environments the experiment will be duplicated in a independent environment. The University of Jaèn smart lab consists of a fully sensorised living room, kitchen, bathroom, and bedroom. The array of sensor technology includes PIR sensors, contact sensors, static cameras, and a smart floor. The results from the duplicated experiment were then compared to the UU results to determine if the level of performance could be replicated, a qualitative assessment was also carried out regarding the ease of installation to a new environment.

### Machine vision system

As wearable devices are traditionally ‘resource poor’ in comparison with contemporary server hardware [10] Google Glass is responsible for capturing the video stream and delivery of reminders/notifications only. The image processing is offloaded to a server via Real Time Streaming Protocol (RTSP) for processing (Fig. 1), thus decreasing the time taken for object detection and for the appropriate response to be given, along with increasing battery life on the Glass platform. Ha et al. carried out a comparison of an assistive application (OCR – Optical Character Recognition), they compared the performance and energy usable of performing the task via on board Google Glass processing in comparision with offloading the processing to a server via a real time stream from Google Glass [10]. Their results are shown in Table 1.

**Table 1:** Comparison of offloading *vs.* on-board processing. Mean over five runs, standard deviation showing in parentheses [10]

Metric	On-Board	Offloading
Per-Image Energy	12.84 (0.36)	1.14 (0.11)
Per-Image Speed	10.49 (0.23)	1.28 (0.12)

As can be seen from Table 1 there is almost a order of magnitude difference in both speed and energy used in offloading compared to on-board processing. Google Glass offers a 2.1V 570mAh (7560 Joule) battery, this equates to an 11 minute battery life when performing on-board processing and an 111 minute battery life when offloading to a server, along with an decrease in the processing time needed to perform recognition. Battery life can be further extended with external battery packs, however, with the current rate of advance in battery technology the battery life of future generations of wearable devices will be less of a challenge.

**Fig. 2:**
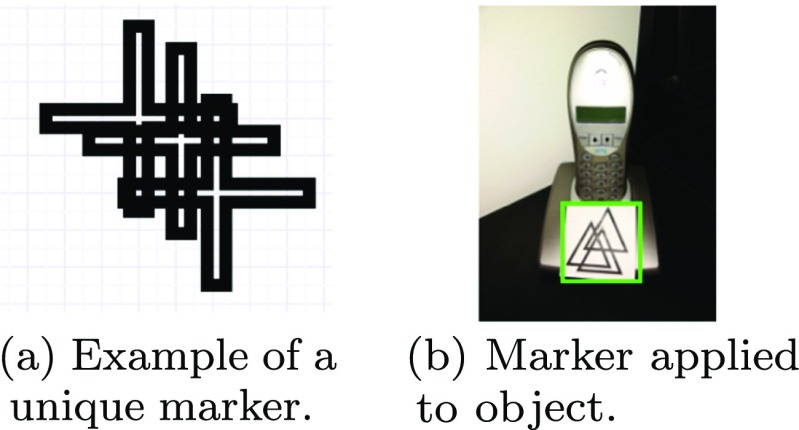
Image (**a**) is an example of the markers used, Image (**b**) shows how the marker is applied to an object of interest, in this case a telephone

To aid in the correct identification of objects unique markers where applied to the objects of interest, as shown in Fig. 2a. This allows a custom identifier to be placed on each marker to distinguish between objects, as shown in Fig. 2b. The unique markers are learnt during a pre-processing stage where the ORB feature points are detected and stored.

The use of markers also reduces some of the issues traditionally faced when performing object recognition, such as variations between the same objects – *i.e.* different models of appliances. Furthermore this also alleviates the problem of distinguishing between multiple identical objects in close proximity, such as kitchen cupboards/drawers [7].

The experiment was carried out using the OpenCV library on an Intel Core2Quad (Q9950) 2.83GHz machine, the video was transmitted at a resolution of 640 x 480 by Google Glass at 20fps. Due to the processing limitations of Google Glass a variable lag (<3s) was introduced on the stream. The lag was due to the Glass’s efforts to lower the operating temperature which it achieves by reducing the clock speed of the CPU [10]. The CPU can be set to four frequencies – 300Mhz, 600Mhz, 800MHz, and 1GHz. At high temperatures the Glass firmware limits the CPU to 600Mhz or 300MHz in order to cool down via power reduction [16].

### ORB feature pints and descriptors

The chosen method of detection and extraction of feature points and descriptors is the ORB (Oriented Features from Accelerated Segment Test and Rotated Binary Robust Independent Elementary Features) keypoint detector/extractor which was developed by Rublee et al. [26]. The ORB algorithm uses FAST (Features from Accelerated Segment Test) in pyramids in order to detect stable key-points and selects the strongest features using FAST. ORB implements a simple method of corner detection, the intensity centroid as defined by Rosin [25]. ORB features are invariant to rotation and scale, resulting in a very fast recogniser which is robust to viewpoint invariance [17], being faster than both SIFT and SURF based algorithms while still maintaining accuracy [6]. Previous studies have shown that a strength of ORB is it’s ability to accommodate low brightness conditions [8], this is in part to ORB implementing the Harris Corner Detection algorithm which has been shown to be robust to low brightness conditions [22].

### K-nearest neighbour matching

A K-Nearest Neighbour (KNN) algorithm is used to match the feature points to determine if an object is present. A simple version of an KNN is used – a Brute-Force matcher. While a Brute-Force matcher is one of the worst performing matchers in terms of time taken to establish a match (detection time as implemented is still less than one second) it is also the best performer in terms of accurately identifying the correct matches as found in [3] which benchmarked multiple algorithms for the purposes of image matching. A formal representation of a KNN algorithm finds the *K* closest (similar) features to a query feature among *N* points in a d-dimensional feature space [30]. Within this implementation the Brute-Force matcher is used to compare feature points for matching pairs, for each feature in the object the matcher finds the closest feature in the scene by trying each one. The similarity between two pairs is represented by Norm Hamming distance. A minimum Hamming distance is set to ensure that only good matches are selected. A match is considered good when the distance is less than three times the minimum Hamming distance set, a brief overview of the process of setting the minimum and maximum distance along with the good match selection pseudo-code is presented in Algorithm 1.

**Figure:**
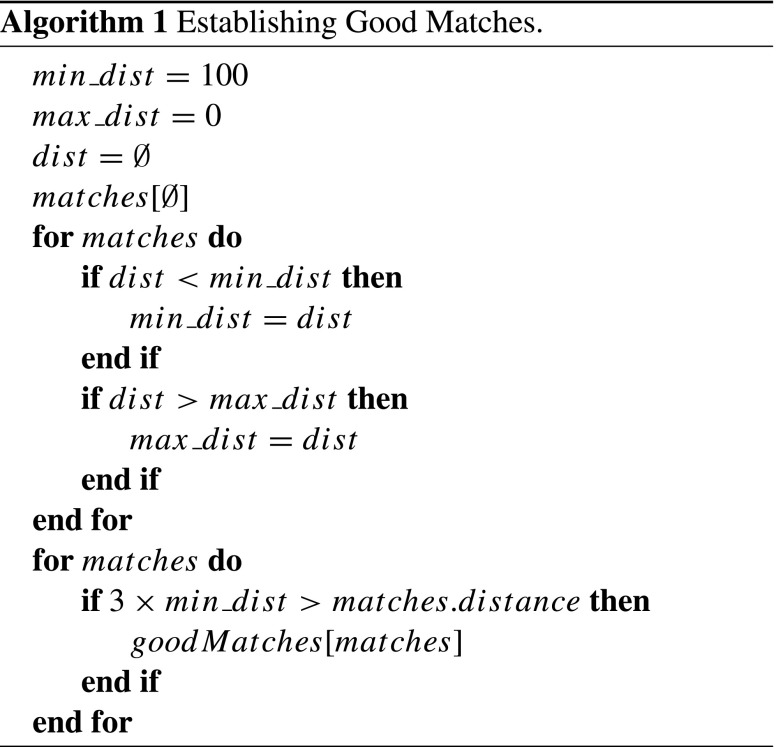

In order to dismiss the number of False Positives (FP – where an object is determined to be present when it is not) reported by the system a two stage filter was used. For the first stage the homography was used as a model for correct matches (‘Keypoint Match Threshold’ in Fig. 1). The number of inliers that contributed to the homography were determined and compared against a threshold value, if the number of inliers match or exceed this value then it is passed onto the second stage. The second stage is a Vote Function where any further FP that have passed through the first stage are removed.A batch of frames (three in this implementation) are processed, the object most likely to be present in each frame is determined and stored. Once the most likely object for each frame has been determined a vote count is performed. Once this count passes a pre-determined threshold value the most likely object is determined to be present.The pseudo-code for the second stage filter can be seen in Algorithm 3.3.

**Figure:**
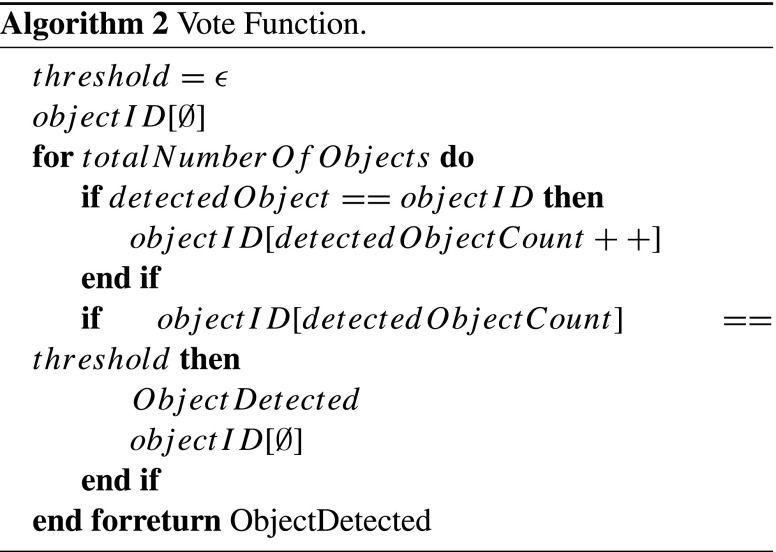

### Contact sensors

Dense sensor placement have also been used as a benchmark in order to provide a comparison with the machine vision system. This consists of TyneTec binary contact sensors that were placed on the same objects that also have a unique machine vision marker placed on them. There was a total of 14 TyneTec sensors which uploaded events to a MySQL database for retrieval. All components of the system were time synced with a MySQL server in order to ensure that the events were synchronised.

### Experiment routines

A range of activities were carried out that were representative of daily routines, with the goal of recognising the component locations that consist each activity. If prepare/drink water is taken as an example activity, then the component locations would be the kitchen door, the cup cupboard, the tap, and then finally the kitchen door again. Three routines were created, the first containing ten activities and the remaining two containing eleven activities. These ranged from simple activities such as drinking a glass of water to more complex activities, such as preparing hot food. The activities are presented in Table 2, with the full routines presented in Table 3.

**Table 2:** Full list of activities that were performed during the three routines

	Full Activity List
1.1	Prepare/drink water
1.2	Prepare/drink tea
1.3	Prepare/drink hot chocolate
1.4	Prepare/drink milk
2	Make/receive phone call
3.1	Prepare/eat cold meal
3.2	Prepare/eat hot meal
4	Watch TV
5	Wash dishes

**Table 3:** Breakdown of activities that took place in each routine

Routine 1 (R1)	Routine 2 (R2)	Routine 3 (R3)
1.3	1.4	1.3
1.1	3.1	1.1
3.2	1.1	2
5	2	3.2
4	1.1	1.1
1.1	1.2	4
4	4	1.2
3.1	3.2	4
5	5	3.1
1.1	4	5
N/A	1.1	1.4

These routines were performed under the same lighting conditions in order to minimise any potential discrepancy between identical activities in differing routines. In order to ensure the accuracy of the machine vision and binary sensor location systems, the ground truth was obtained from a time stamped video. The inhabitant’s location reported from the location systems where then compared to the ground truth from the video.

### Experiment duplication

In order to assess the viability in applying the proposed solution to multiple environments the aforementioned routines were carried out in a second location, the smart lab of the University of Jaèn, Spain. Ceiling lighting and window blinds were used to control the lighting conditions. Additionally, activities remained the same within each routine along with both the markers and wearable sensor, the only variable being the environmental layout. Ground truth was gathered from manually annotated video data in order to ensure the accuracy of the vision system. As it is the viability of the vision system that is of interest only the vision results will be compared between the results of the experiment in UU and Jaèn.

## Results

This Section describes the results of the machine vision localisation system, along with details on the results from the dense sensor system when compared with the ground truth from the annotated video data. Due to the high number of True Negatives (TN), over twenty thousand, from the machine vision system a skewed dataset was produced. Due to this accuracy was determined by measuring recall, precision, and F-Measure. These will be focused on to avoid the high number of TN giving an incorrect weighting to the results.

The results from the machine vision system at Ulster University (UU) are presented in Tables 4, 5 and 6, and the results from the binary contact sensors are presented in Tables 7 and 8. As shown in Table 5 there is a total of 32 False Negatives (FN – where an object was present but not detected), the majority of these (11) were due to corruption within the video frame during transmission, the rest of the FN’s where due to varying reasons, such as missing frames (Table 9).

**Table 4:** Results of Recall, Precision, and F-Measure for the machine vision based system – UU

Routine	Total Events	Recall	Precision	F-Measure
R1	58	0.74	0.98	0.84
R2	56	0.88	0.94	0.91
R3	61	0.84	0.96	0.89
Total	175	0.82	0.96	0.88

**Table 5:** Breakdown of machine vision sensor classification outcomes including TP, FN, and FP – UU

Routine	Total Events	TP	FN	FP
R1	58	43	15	1
R2	56	49	7	3
R3	61	51	10	2
Total	175	143	32	6

**Table 6:** Breakdown of machine vision sensor classification outcomes including TP, FN, and FP – Jaèn

Routine	Total Events	TP	FN	FP
R1	58	39	19	1
R2	56	38	18	1
R3	61	39	22	1
Total	175	116	59	3

**Table 7:** Results of Recall, Precision, and F-Measure for the dense sensor based system

Routine	Total Events	Recall	Precision	F-Measure
R1	58	1.00	1.00	1.00
R2	56	0.93	1.00	0.96
R3	61	0.90	1.00	0.95
Total	175	0.94	1.00	0.97

**Table 8:** Breakdown of dense sensor classification outcomes including TP, FN, and FP

Routine	Total Events	TP	FN	FP
R1	58	58	0	0
R2	56	52	4	0
R3	61	55	6	0
Total	175	165	10	0

**Table 9:** Results of Recall, Precision, and F-Measure for the machine vision based system – Jaén

Routine	Total Events	Recall	Precision	F-Measure
R1	58	0.67	0.98	0.80
R2	56	0.68	0.97	0.80
R3	61	0.64	0.98	0.77
Total	175	0.66	0.97	0.79

Table 10 presents a breakdown of the missed events by the machine vision system at UU along with an attempted explanation as to why the events were missed. It should also be noted that eight contact sensor events were missed due to a battery failure part way through the experiment; there were three such events in R2 and five events in R3 that were missed.

**Table 10:** A breakdown of FN machine vision events – UU

Cause	FN
Corrupt frame	16
Other	8
Unknown	8
Total	32

Tables 9 and 11 presents the machine vision results from the lab in Jaèn. As can be seen from Tables 9 and 4 there is reduction of the average Recall and F-measure by 0.16 and 0.09 respectively with a rise in Precision of 0.01.

**Table 11:** A breakdown of FN machine vision events – Jaén

Cause	FN
Unfocused	47
Other	12
Total	59

As shown in Table 6 there is a total of 59 FN, with the majority of these (47) being due to the camera autofocus not being able to correctly focus on the object. Table 11 presents a breakdown of the FN causes that effected the vision system at Jaèn.

## Discussion

While the binary contact sensors provided more accurate results this does not fully demonstrate the additional advantages the machine vision system provides over dense sensor placement. One of the key advantages this method offers is that interaction with an object is not required in order to determine the occupant’s location within the environment which can offer a more timely location update compared to dense sensor placement. In the experiment the occupant’s location was reported before they had interacted with the object thus offering a more timely update. Also if the occupant became confused or decided not to use the object their location would still be captured. This would have otherwise been lost in a traditional sensor based smart environment. Another potential advantage is that of multiple occupancy, as each occupant will use a wearable device it would be possible to locate each occupant within the environment and to infer their activity from their own first person view. Nevertheless, this is working under the assumption that only the occupants of the environment will require support, as any visitors will not have a wearable device. If any sensor activity is detected without a corresponding machine vision event then it would be assumed that the visitors have activated a sensor and thus that event should be ignored.

This paper also assesses the viability to applying this solution to other environments, as occupants generally have to be supported within their own home this is an important aspect of developing a solution to that of AAL in the home. The proposed system offers reduced financial costs in terms of initial equipment purchase and maintenance, along with a reduction in the invasiveness for the installation compared to traditional indoor localisation methods. The issue of multiple occupancy is also partially address as this solution allows individual support to be given to each occupant as they have a unique first-person view of the environment, this does however assume that only the occupants require support and that any visitors to the environment can be assumed to not require any assistance. This will allow support to be given in the form of notifications/reminders in order to assist with Activities of Daily Living (ADL). This solution aims to improve context aware support through the localisation of objects within a smart environment.

The results from the experiment in the University of Jaèn offer an insight into the viability of applying the system to other environments. As the markers are placed on common objects that are ubiquitous to every home environment the markers used in the UU experiment were able to be directly used in when recreating the experiment in Jaèn with no modification. This allowed a simple and fast set up time (∼five minutes) compared to traditional methods such as dense sensor placement or the fitment of static cameras [15, 32]. Due to the small nature of the dataset missed events have a larger impact resulting in a drop in recall and F-Measure, however the Precision increased. Despite this the results show that the method is viable across multiple environments, although the creation of a larger dataset is warranted to gain a more accurate picture of the performance.

One aspect of AAL that must be taken into consideration is the acquisition and maintenance costs of implementing a sensorised environment. A large network of embedded sensors is normally required which results in a system that is costly to maintain, relatively obtrusive (as sensors are required on every intractable object), and sensitive to the performance of the sensors [2]. Table 12 shows the costs involved in implementing both dense sensor and fixed video camera systems within a household. As can been seen from the Table 12 there is a high financial cost involved in the purchase and installation of traditional methods of indoor localisation. While a DIY installation goes a long way to reduce these costs (Control4 price is reduced by $70,000 from the professional installation), it must be considered that as the users that would benefit from such as system may not be physically or mentally fit to carry out such an intensive installation. An additional advantage towards the proposed system, and vision systems in general, is that generic hardware can be used for multiple applications to aid in AAL [2].

**Table 12:** A breakdown of costs with associated sensor platforms [1]

System	Cost	Installation
Elk M1	$5,000	DIY
Lagotek	$5,000	DIY
Control4	$50,000	DIY
X10	$300	DIY
Creston	$60,000	Professional
Control4	$120,000	Professional
EIB Instabus	$13,500	Professional

## Conclusion

A method of indoor localisation has been presented utilising a wearable camera to determine location based upon objects viewed within a scene. This was compared with a common method of indoor localisation (dense sensor placement) employing annotated video data as the ground truth. Thus supporting the hypothesis that the use of a single wearable camera allows inhabitant tracking within an environment with the goal of determining location. While the machine vision results were not as accurate as the dense sensor placement, they demonstrated that the proposed method is viable and offers other secondary advantages that are unique to this method, such as the first person view and lack of required interaction.

The work presented demonstrates the viability of applying the solution to differing environments. The performance of the system at Jaèn was comparable with the previous experiment carried out at UU. With the Jaèn experiment showing an average recall, precision, and F-measure of 0.66, 0.97, and 0.79 respectively in comparison to the UU recall, precision, and F-measure results of 0.82, 0.96, and 0.88 respectively. The duplication of the experiment in Jaèn established the viability of applying the solution to multiple environments which has been shown to be a challenge within the domain of AAL, as can be seen in Section 2. The lack of training, use of common objects and hardware can be attributed to this success.

However, there are some limitations to using such as static approach to storing the objects location within a knowledge base, such as objects being moved or certain objects that may not have a static location, for example personal devices. Another limitations inherent with wearable camera solutions is that they rely on an ‘always-wear’ approach were the system is reliant on the user to remember to put the Glass on in the morning. This is somewhat mitigated that 74 % of the adult population wear corrective lenses [29] and with the ability to insert prescription lenses into Google Glass it could replace their normal glasses to try and avail of their daily routine of wearing glasses. Future work will involve determining activity based on the objects located within the field of view, through the use of a rule-based system in order to provide support for those activities through the use of a multi-agent system with each agent governing an activity in order to provide specific support for said activity. The long term aspiration of this system is to assist those in cognitive decline with their ADL, such as in the event the occupant has became confused with a task part way through, for example making a meal; assistance could then be provided to allow the continuation of the task.
